# Gyeji-tang water extract exerts anti-inflammatory activity through inhibition of ERK and NF-κB pathways in lipopolysaccharide-stimulated RAW 264.7 cells

**DOI:** 10.1186/s12906-016-1366-8

**Published:** 2016-10-12

**Authors:** Sae-Rom Yoo, Yeji Kim, Mee-Young Lee, Ohn-Soon Kim, Chang-Seob Seo, Hyeun-Kyoo Shin, Soo-Jin Jeong

**Affiliations:** 1 K-herb Research Center, Korea Institute of Oriental Medicine, Daejeon, 34054 Republic of Korea; 2 KM Convergence Research Division, Korea Institute of Oriental Medicine, 1672 Yuseong-daero, Yuseong-gu, Daejeon, 34054 Republic of Korea; 3 Mucosal Immunology Laboratory, Department of Convergence Medicine, University of Ulsan College of Medicine/Asan Medical Center, Seoul, Republic of Korea; 4 Korean Medicine Life Science, University of Science and Technology, Daejeon, Republic of Korea

**Keywords:** Gyeji-tang, Supression of inflammation, Macrophage, Extracellular signal-regulated kinase, Nuclear factor kappa B

## Abstract

**Background:**

Gyeji-tang (GJT, Guizhi Tang in Chinese, Keishi-to in Japanese) is a traditional herbal decoction composed of 5 medicinal herbs. GJT has been used to treat the common cold, headaches, and fever in Asian countries including Korea, China, and Japan. In the present study, we investigated the inhibitory effect of a water extract of GJT on inflammatory response using the murine macrophage cell line, RAW 264.7.

**Methods:**

RAW 264.7 macrophages were treated with lipopolysaccharide (LPS) to upregulate inflammatory genes. Cells were pretreated with various concentrations of GJT for 4 h and stimulated with LPS for an additional 20 h. Productions of tumor necrosis factor-alpha (TNF-α), interleukin-6 (IL-6), cyclooxygenase-2 (COX-2), and prostaglandin E_2_ (PGE_2_) were measured by enzyme-linked immunosorbent assays (ELISAs). Protein expressions of heme oxygenase (HO)-1, extracellular signal-regulated kinase (ERK), and nuclear factor kappa-B (NF-κB) were analyzed by immunoblotting.

**Results:**

Treatment with the GJT extract enhanced expression of HO-1 in macrophages without cytotoxicity. GJT extract significantly inhibited proinflammatory cytokines TNF-α and IL-6 in LPS-stimulated cells. GJT suppressed LPS-induced COX-2 expression, leading to a decrease in COX-2-derived PGE_2_ level. In addition, GJT extract prevented phosphorylation of ERK and NF-κB translocalization to the nucleus in LPS-treated RAW 264.7 cells.

**Conclusion:**

These data suggest that GJT has anti-inflammatory possibly through blocking ERK and NF-κB signaling pathways.

## Background

Inflammation is a tissue protective response against potentially harmful stimuli such as bacteria, damaged cells, or irritants [1, 2]. Chronic inflammation is associated with pathogenesis of the various diseases such as cancer, atherosclerosis, rheumatoid arthritis, and type 2 diabetes. Macrophages are significantly involved in the initiation and maintenance of the inflammatory process through secretion of inflammatory cytokines [3]. Blocking proinflammatory cytokines such as tumor necrosis factor-alpha (TNF-α) or interleukin-6 (IL-6) is considered as an attractive therapeutic approach to inflammation [4, 5]. In addition to inflammatory cytokines, prostaglandin E2 (PGE_2_) as a proinflammatory mediators plays an important role in inflammatory response. PGE_2_ is synthesized by cyclooxygenase-2 (COX-2) that is activated by proinflammatory stimuli such as cytokines, endotoxin, or growth factors [6]. These proinflammatory regulators can be controlled by several intracellular molecular pathways such as those involving mitogen-activated protein kinases (MAPKs) and nuclear factor kappa-B (NF-κB) [7]. Chronic inflammation and its related diseases are associated with the oxidative stress that generates proinflammatory mediators and cytokines. The antioxidative enzyme heme oxygenase 1 (HO-1) has anti-inflammatory properties and is therefore thought to be a potential molecular target for treating inflammatory diseases [8, 9]. In macrophages, activation of HO-1 inhibits cytokine secretion [10, 11] and suppresses COX-2 expression [12].

Gyeji-tang (GJT, Guizhi Tang in Chinese, Keishi-to in Japanese), a traditional Korean medicine, has been used to treat cold, headache, and fever in Asian countries including Korea, China and Japan [13]. GJT consists five herbs. However, the scientific evidence to support antiinflammatory effect of GJT is rare. To date, several groups reported effects of GJT on pancreatic acinar cell injury [14], diabetes mellitus [15], and Guizhi decoction syndrome [16]. In the present study, we investigated anti-inflammatory mechanisms of GJT water extract using lipopolysaccharide (LPS)-stimulated RAW 264.7 murine macrophages. Inhibitory effects of GJT against the inflammatory response was elucidated by measuring production of TNF-α, IL-6 and PGE_2_, and analyzing MAPK and NF-κB pathways, and HO-1 expression.

## Methods

### Plant materials

The 5 medicinal herbs comprising GJT were purchased from Kwang Myung Dang Medicinal Herbs (Ulsan, Korea) as shown in Table 1. The taxonomic authenticity of these medicinal herbs was confirmed by Prof. Je Hyun Lee, Dongguk University, Gyeongju, Korea. Voucher specimen (2012–KE46–1 through KE46–5) have been deposited at the K-herb Research Center, Korea Institute of Oriental Medicine.

**Table 1:** Herbal composition of GJT

Herbal medicine	Scientific name	Origin	Amount (g)
Cinnamomi Ramulus	*Cinnamomum cassia*	Vietnam	11.25
Paeoniae Radix	*Paeonia lactiflora*	Uiseong, Korea	7.50
Glycyrrhizae Radix et Rhizoma	*Glycyrrhiza uralensis*	China	3.75
Zingiberis Rhizoma Crudus	*Zingiber officinale*	Yeongcheon, Korea	3.75
Zizyphi Fructus	*Zizyphus jujuba*	Yeongcheon, Korea	3.75
Total amount			30.00

### Preparation of GJT water extract

Five medicinal herbs was mixed as shown in Table 1 (total 5.0 kg; 30.0 g × 166.7) and extracted in a 10-fold mass of water at 100 °C for 2 h under pressure (1 kgf/cm^2^) using an electric extractor (COSMOS-660; Kyung Seo Machine Co., Incheon, Korea). The water extract was then filtered through a standard sieve (No. 270, 53 μm; Chung Gye Sang Gong Sa, Seoul, Korea). The solution was freezing-drying to give a powder using PVT100 freeze dryer (IlShinBioBase, Yangju, Korea). The yield of the GJT water extract was 9.75 % (487.5 g).

### Cell culture

The murine macrophage cell line, RAW 264.7, was obtained from the American Type Culture Collection (Rockville, MD). The cells were cultured in Dulbecco’s modified Eagle’s medium (Gibco Inc., Grand Island, NY) supplemented with 5.5 % heat-inactivated fetal bovine serum (Gibco Inc.), penicillin (100 U/mL), and streptomycin (100 μg/mL) under an atmosphere of 5 % CO_2_ in an incubator at 37 °C.

### Cytotoxicity assay

Cell viability assay was performed to determine the cytotoxicity of GJT using a Cell Counting Kit-8 (CCK-8; Dojindo, Kumamoto, Japan). Cells were plated into a 96-well microplates at 3 × 10^3^ cells/well and treated with various concentrations of GJT for 24 h. After incubation with CCK-8 reagent for 4 h, optical density (OD) at 450 nm was measured by using a Benchmark plus microplate reader (Bio-Rad Laboratories, Hercules, CA). Cell viability was calculated using the following equation:

$$ \mathrm{Cell}\ \mathrm{viability}\ \left(\%\right)=\frac{\mathrm{Mean}\ \mathrm{O}\mathrm{D}\ \mathrm{in}\ \mathrm{G}\mathrm{J}\mathrm{T}\ \mathrm{treated}\ \mathrm{cells}}{\mathrm{Mean}\ \mathrm{O}\mathrm{D}\ \mathrm{in}\ \mathrm{untreated}\ \mathrm{cells}}\times 100 $$

### ELISAs for TNF-α, IL-6, and PGE_2_

Cells were pretreated with various concentrations of GJT for 4 h and stimulated with LPS (1 μg/mL) for an additional 20 h. Production of TNF-α, IL-6, and PGE_2_ in the culture supernatants was measured using commercial ELISA kits from R&D systems (Minneapolis, MN), BD Biosciences (Mountain View, CA), and Cayman Chemical Co. (Ann Arbor, MI), respectively.

### Reverse transcription-polymerase chain reaction (RT-PCR)

Total RNA was extracted using Trizol reagent (Invitrogen Life Sciences, Carlsbad, CA, USA) according to the manufacturer’s instructions. cDNA was synthesized from 1 $\mu$g of total RNA using an iScript cDNA synthesis kit (Bio-Rad Laboratories, Hercules, CA, USA) and subjected to PCR reactions with rTaq DNA polymerase (ELPIS Biotech Inc., Daejeon, South Korea). The relative expression of COX-2 was analyzed using β-actin as an internal control. The primer sequence for *COX-1* was forward 5′-AGG AGA TGG CTG CTG AGT TGG-3′ and reverse 5′-AAT CTG ACT TTC TGA GTT GCC-3′, *COX-2* was forward 5′-GTA TCA GAA CCG CAT TGC CTC TGA-3′ and reverse 5′-CGG CTT CCA GTA TTG AGG AGA ACA GAT-3′, and *β-actin* was forward 5′-ACC GTG AAA AGA TGA CCC AG-3′ and reverse 5′-TAC GGA TGA CAA CGT CAC AC-3′. The PCR conditions were 25 cycles of 94 °C for 30 s, 57 °C (β-actin) or 59 °C for 1 min, and 72 °C for 1.5 min. The amplification products were then separated by electrophoresis on 1 % agarose gels and detected using a Molecular Imager Gel Doc XR System (Bio-Rad Laboratories, Hercules, CA, USA).

### Western blotting

Whole cell extract was prepared by suspending cells in an extraction lysis buffer (Sigma-Aldrich, St. Louis, MO) containing protease inhibitor cocktail (Roche Applied Science, Indianapolis, IN). Nuclear extract was isolated using NE-PER Nuclear and Cytoplasmic Extraction reagents (Thermo Scientific, Rockford, IL) according to the manufacturer’s protocol. Protein concentrations in the extracts were determined using a Bio-Rad Protein Assay reagent (Bio-Rad, Hercules, CA). Equal amount of cell extract proteins (30 μg) were resolved by 4 %–20 % sodium dodecyl sulfate-polyacrylamide gel electrophoresis (SDS-PAGE) and transferred to a polyvinylidene fluoridemembranes. The membrane was incubated with blocking solution (5 % skim milk in Tris-buffered saline containing Tween 20 (TBST), followed by an overnight incubation at 4 °C with the appropriate primary antibodies; anti-phospho-p38 MAPK, anti-phospho-ERK, anti-phospho-JNK (Cell Signaling, Danvers, MA), HO-1 (Abcam, Boston, MA), NF-κB p65, HO-1, and β-actin (Santa Cruz Biotechnology, Dallas, TX). The membranes were washed three times with TBST, and then incubated with a horseradish peroxidase (HRP)-conjugated secondary antibody (Jackson ImmunoResearch, West Grove, PA) for 1 h at room temperature. The membranes were washed three times with TBST again, and then developed using an enhanced chemiluminescence kit (Thermo Scientific). Image capture was performed using a Chemi-Doc XRS system (Bio-Rad).

### Immunofluorescence staining

Cells were plated onto poly-l-lysine coated glass slides and fixed in 4 % (v/v) methanol free formaldehyde solution (pH 7.4) at 4 °C for 25 min. The cells were permeabilized in 0.2 % (w/v) Triton X-100, blocked in 5 % (w/v) bovine serum albumin (BSA) in humidified chamber, followed by immunostaining with NF-κB p65 antibody (Santa Cruz Biotechnology, Santa Cruz, CA) and Texas Red-conjugated secondary antibody. The cells were mounted with mounting medium with coverslips with a mounting medium containing DAPI (Vector Laboratories, Inc, Burlingame, CA) and visualized under an FlouviewFV10i confocal microscope (Olympus, Tokyo, Japan).

### Chemicals

The reference standards, gallic acid, benzoic acid, and coumarin were purchased from Sigma-Aldrich (St. Louis, MO, USA). Albiflorin, paeoniflorin, liquiritin, cinnamic acid, cinnamaldehyde, glycyrrhizin, and 6-gingerol were purchased from Wako (Osaka, Japan). Benzoylpaeoniflorin and liquiritin apioside were purchased from Biopurify Phytochemicals (Chengdu, China) and Shanghai Sunny Biotech (Shanghai, China). A standard stock solution of these components were dissolved in methanol at concentrations of 1.0 mg/mL. For HPLC analysis of the GJT extract, 100 mg of lyophilized GJT extract was dissolved in 20 mL of distilled water and then the solution was diluted to 10-fold for quantitative analysis of paeoniflorin. Solutions were filtered through a SmartPor GHP 0.2 μm syringe filter (Woong Ki Science Co., Seoul, Korea) before application to the column.

### HPLC analysis of GJT

Quantitative analysis of 12 compounds present in the GJT extract was performed using a Shimadzu LC-20A HPLC system (Shimadzu Co., Kyoto, Japan) consisting of a solvent delivery unit, an on-line degasser, a column oven, an autosampler, and a PDA detector. The data were acquired and processed by LabSolution software (version 5.54 SP3l Shimadzu Co.). The analytical column used was a SunFire C_18_ (250 × 4.6 mm; particle size 5 μm, Waters, Milford, MA, USA) and was maintained at 40 °C. The mobile phases consisted of water (A) and acetonitrile (B), which were both containing 0.1 % (v/v) formic acid. The gradient flow was as follows: 5 % – 60 % B for 0–30 min, 60 % – 100 % B for 30–40 min, 100 % B for 40–45 min, and 100–5 % B for 45–50 min. The flow-rate was 1.0 mL/min and injection volume was 10 μL.

### Statistical analysis

The data are expressed as the mean ± SEM. Data were analyzed using one-way analysis of variance and Dunnett’s multiple comparisons test. *P* < 0.05 was considered significant.

## Results

### GJT enhances HO-1 expression in RAW264.7 cells

**Fig. 1:**
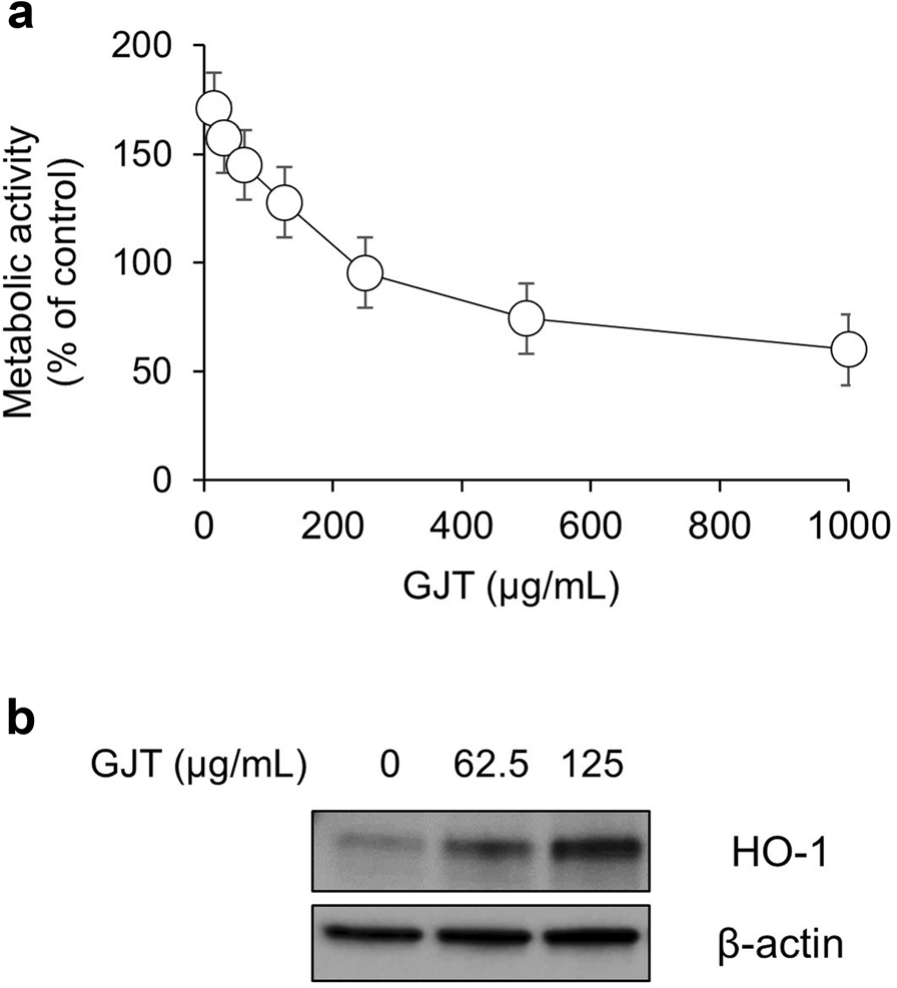
Effects of GJT on the cell viability and HO-1 expression in RAW264.7 macrophages. **a** Cells were treated with various concentrations of GJT (31.25, 62.5, 125, 250, 500, or 1000 μg/mL) for 24 h. Cell viability was measured by CCK assay. **b** Cells were treated with GJT (0, 62.5, or 125 μg/mL) for 24 h. Whole cell lysates were prepared and subjected to immunoblotting for HO-1

Macrophages play an important role in the process of inflammation including its initiation, maintenance, and resolution through the production of pro- or anti-inflammatory cytokines [3]. In the present study, RAW 264.7 murine macrophages were used to investigate anti-inflammatory effects of the GJT extract. To evaluate the cytotoxicity of GJT against RAW 264.7 cells, we performed a CCK assay. Cells were treated with various concentrations of GJT extract (15.63, 31.25, 62.50, 125.00, 250.00, 500.00, or 1000.00 μg/mL) for 24 h. As shown in Fig. 1a, the cell viability was maintained at 95.38 ± 0.87 % at ≤ 250 μg/mL. However, higher concentrations of GJT extract (500 and 1000 μg/mL) decreased cell viability by 74.28 ± 0.74 % and 59.89 ± 1.05 %, respectively. Subsequent assays in RAW 264.7 cells were conducted in nontoxic concentrations of GJT extract. To investigate the inhibitory effects of GJT extract on inflammation, we initially analyzed HO-1 expression in RAW 264.7 cells. Treatment with GJT extract induced HO-1 expression in a dose-dependent manner at noncytotoxic concentrations (Fig. 1b).

### GJT suppresses TNF-α and IL-6 secretion in LPS-stimulated RAW264.7 cells

**Fig. 2:**
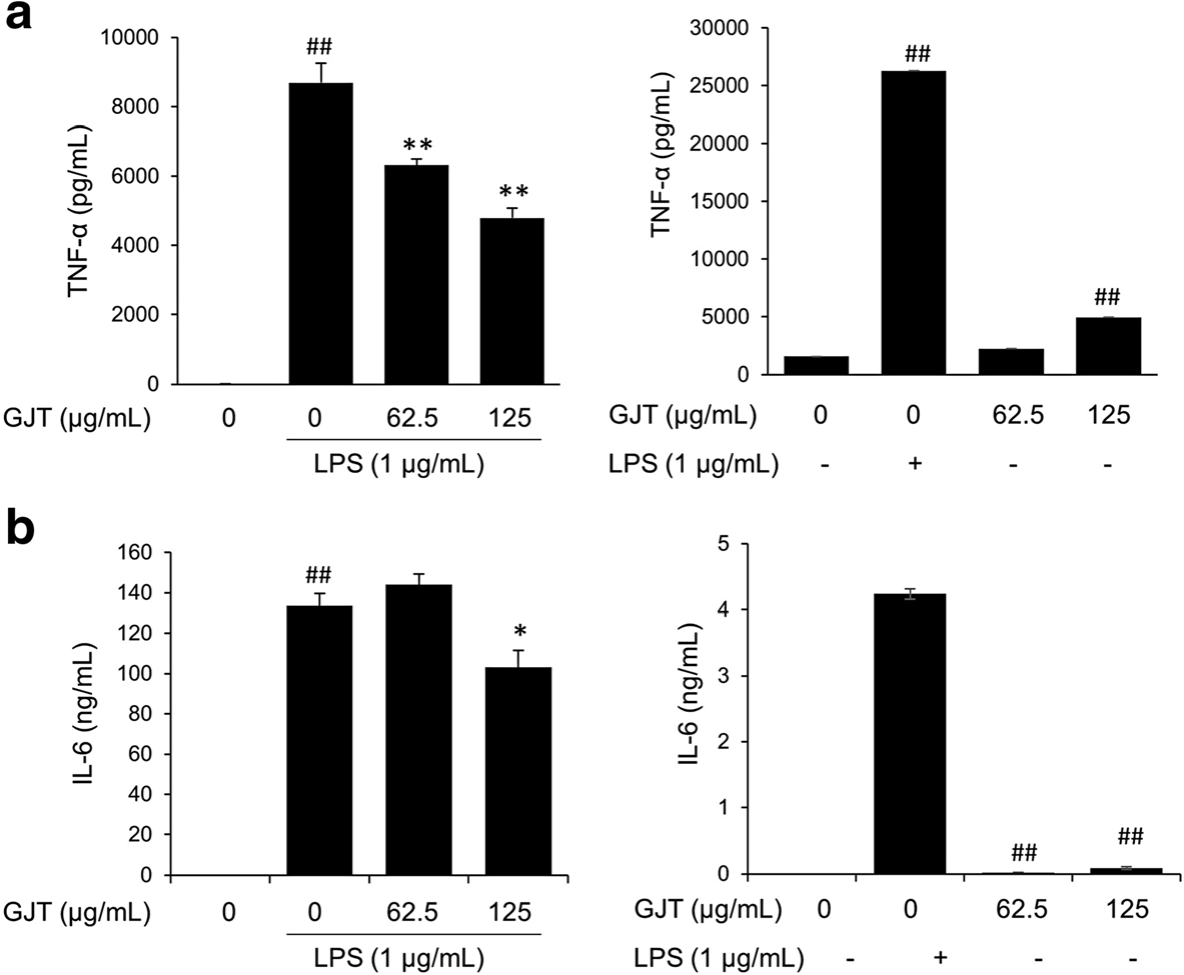
Effects of GJT on production of TNF-α and IL-6 in LPS-stimulated RAW264.7 macrophages. Cells were pretreated with GJT (0, 62.5, or 125 μg/mL) for 4 h and then treated with LPS (1 μg/mL) for 20 h. Supernatants were collected and subjected to ELISAs for **a** TNF-α and **b** IL-6. Bar graphs represent the mean ± SEM from three independent experiments. ^##^ *P* < 0.01 *vs* vehicle control cells, and ^*^ *P* < 0.05 and ^**^ *P* < 0.01 *vs* LPS-treated cells. **c**; untreated control

To examine whether GJT can influence secretion of proinflammatory cytokines TNF-α and IL-6, GJT extracts were added into LPS-stimulated RAW 264.7 cells. LPS significantly enhanced the amounts of secreted TNF-α by 8694.92 ± 560.8 pg/mL compared with untreated control (6.06 ± 6.06 pg/mL). In contrast, GJT significantly reduced TNF-α production in a dose-dependent manner (6318.46 ± 6.06 pg/mL at 62.5 μg/mL and 4790.84 ± 281.17 pg/mL at 125 μg/mL) compared with LPS-stimulated control (Fig. 2a, left panel). In RAW 264.7 cells, LPS stimulation also increased secretion of IL-6 by 133.50 ± 6.08 ng/mL. Treatment with GJT at 125 μg/mL significantly suppressed IL-6 production by 103.10 ng/mL compared with LPS stimulation (Fig. 2b, left panel). Treatment with GJT alone weakly increased levels of TNF-α and IL-6 compared with untreated control (Fig. 2a and b, right panels).

### GJT inhibits PGE_2_ production and COX-2 expression in LPS-stimulated RAW264.7 cells

**Fig. 3:**
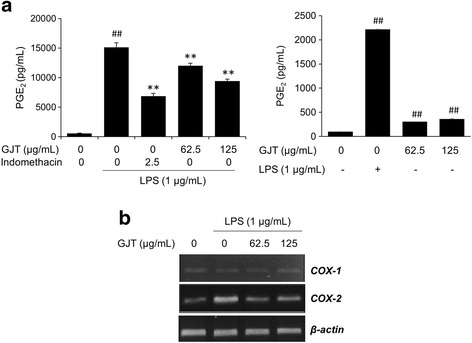
Effects of GJT on PGE_2_ production and COX-2 expression in LPS-stimulated RAW264.7 macrophages. Cells were pretreated with GJT (0, 62.5, or 125 μg/mL) for 4 h and then treated with LPS (1 μg/mL) for 20 h. **a** Supernatants were collected and subjected to ELISAs for PGE_2_. **b** Total RNA was isolated from the cell pellets and subjected to RT-PCR for detecting COX-1 and COX-2 mRNA expression. Levels of COX-1 and COX-2 were adjusted by β-actin expression. Bar graphs represent the mean ± SEM from three independent experiments. ^##^ *P* < 0.01 *vs* vehicle control cells, and ^**^ *P* < 0.01 *vs* LPS-treated cells. **c**; untreated control

PGE_2_ is a key inflammatory mediator synthesized by COX-2 [6]. To support the observed inhibitory effect of GJT on inflammatory response, a PGE_2_ assay was conducted using LPS-stimulated RAW 264.7 cells with or without GJT extract. As shown in Fig. 3a (left panel), LPS stimulation significantly increased production of PGE_2_ by 15149.2 ± 768.01 pg/mL compared with untreated control (552.16 ± 58.82 pg/mL). By contrast, LPS-stimulated PGE_2_ increase was significantly inhibited by GJT at 62.5 and 125 μg/mL by 12032.07 ± 423.16 and 9410.4 ± 315.41 pg/mL, respectively. Treatment with GJT alone showed a marginal increase in the production of PGE_2_compared with untreated cells (Fig. 3a, right panel). Indomethacin used as a positive control also significantly decreased the level of PGE_2_ production by LPS-treated RAW 264.7 cells. Treatment with GJT extract consistently suppressed COX-2, but not COX-1, mRNA expression induced by LPS stimulation (Fig. 3b), indicating the specificity of GJT as a COX-2 inhibitor.

### GJT abrogates ERK phosphorylation in LPS-stimulated RAW264.7 cells

**Fig. 4:**
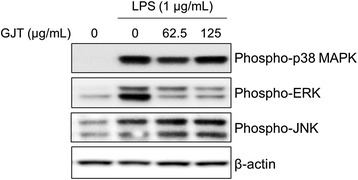
Effects of GJT on activation of the MAPKs in LPS-stimulated RAW264.7 macrophages. Cells were pretreated with GJT (0, 62.5, or 125 μg/mL) for 2 h and the treated with LPS (1 μg/mL) for 15 min. Cell lysates were prepared and subjected to immunoblotting for detecting the phosphorylation of p38 MAPK, ERK, and JNK. **c**; untreated control

MAPK signaling pathways are considered as molecular targets for anti-inflammatory therapy [17]. Therefore, we investigated the effect of GJT on the LPS-mediated phosphorylation of MAPK family members JNK, ERK, and p38 MAPK in RAW 264.7 cells. The cells were pretreated with GJT (0, 62.5, or 125 μg/mL) for 4 h and stimulated with LPS for 15 min. As shown in Fig. 4, LPS stimulation markedly increased levels of phosphorylated p38 MAPK, ERK, and JNK compared with untreated cells. By contrast, treatment with GJT at 62.5 and 125 μg/mL reduced LPS-induced phosphorylation of ERK. However, GJT had no significant effect on JNK and p38 MAPK phosphorylation in LPS-treated RAW 264.7 cells.

### GJT inhibits NF-κB activation in LPS-stimulated RAW264.7 cells

**Fig. 5:**
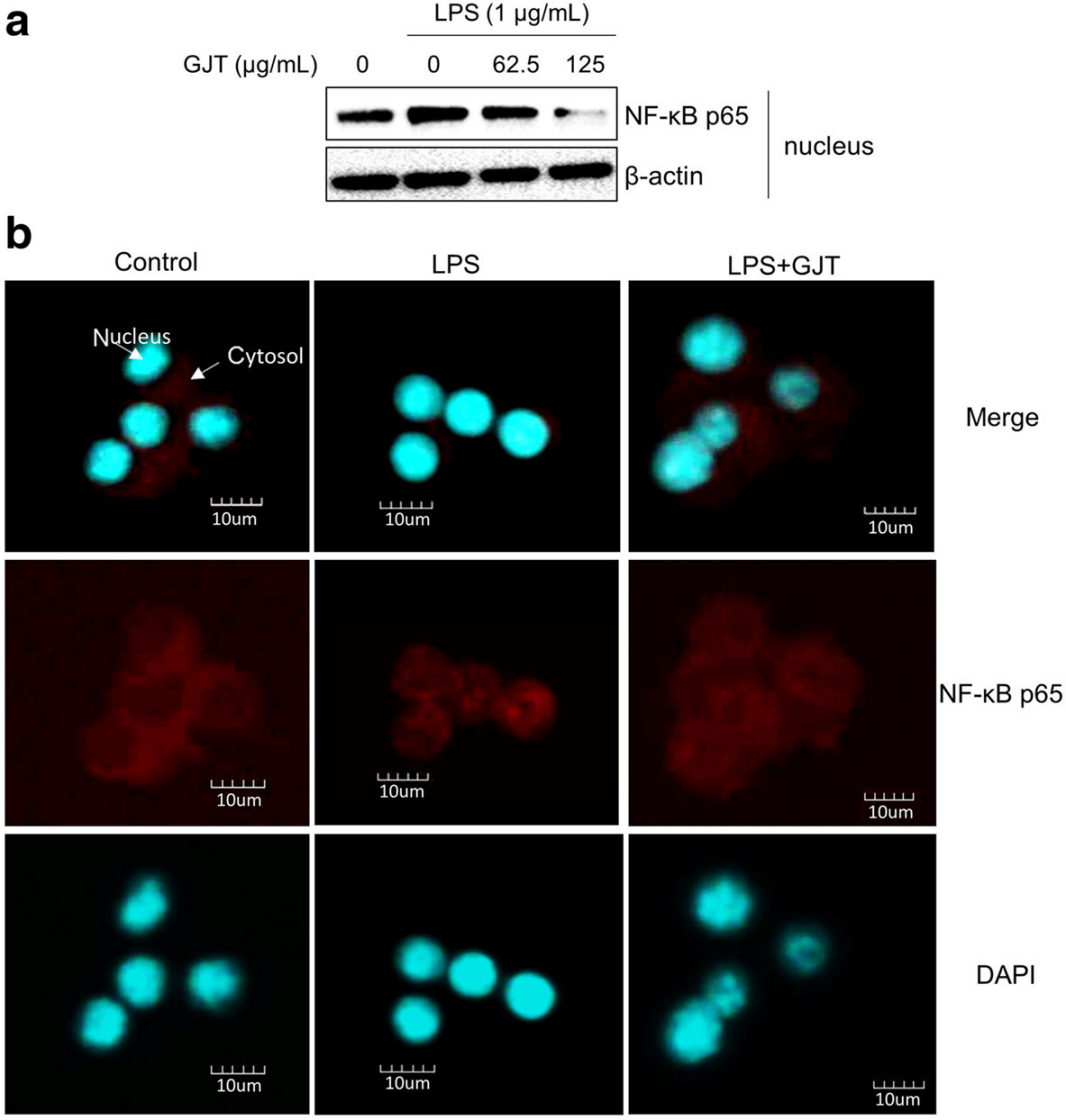
Effects of GJT on activation of NF-κB in LPS-stimulated RAW264.7 macrophages. **a** Cells were pretreated with GJT (0, 62.5, or 125 μg/mL) for 2 h and the treated with LPS (1 μg/mL) for 1 h. Nuclear extracts were prepared and subjected to immunoblotting for NF-κB p65. **b** Cells were pretreated with or without GJT (125 μg/mL) for 2 h and the treated with LPS (1 μg/mL) for 1 h. The cells were fixed in 4 % (v/v) methanol free formaldehyde solution (pH 7.4), stained with anti-NF-κB p65 (*Texas Red*). The immunostained cells were then mounted with medium containing DAPI and visualized under an Olympus FLUOVIEW FV10i confocal microscopy. **c**; untreated control

NF-κB signaling is involved in the development of inflammatory disorders [18]. We observed that GJT extract at 125 μg/mL markedly blocked LPS-stimulated nuclear expression of NF-κB p65 (Fig. 5a). Consistently, immunocytochemistry showed that LPS stimulation induced translocalization of NF-κB p65 from the cytosol into the nucleus whereas with GJT extract blocked the LPS-mediated nuclear translocalization of NF-κB p65 in RAW 264.7 cells (Fig. 5b).

### HPLC analysis of GJT

**Fig. 6:**
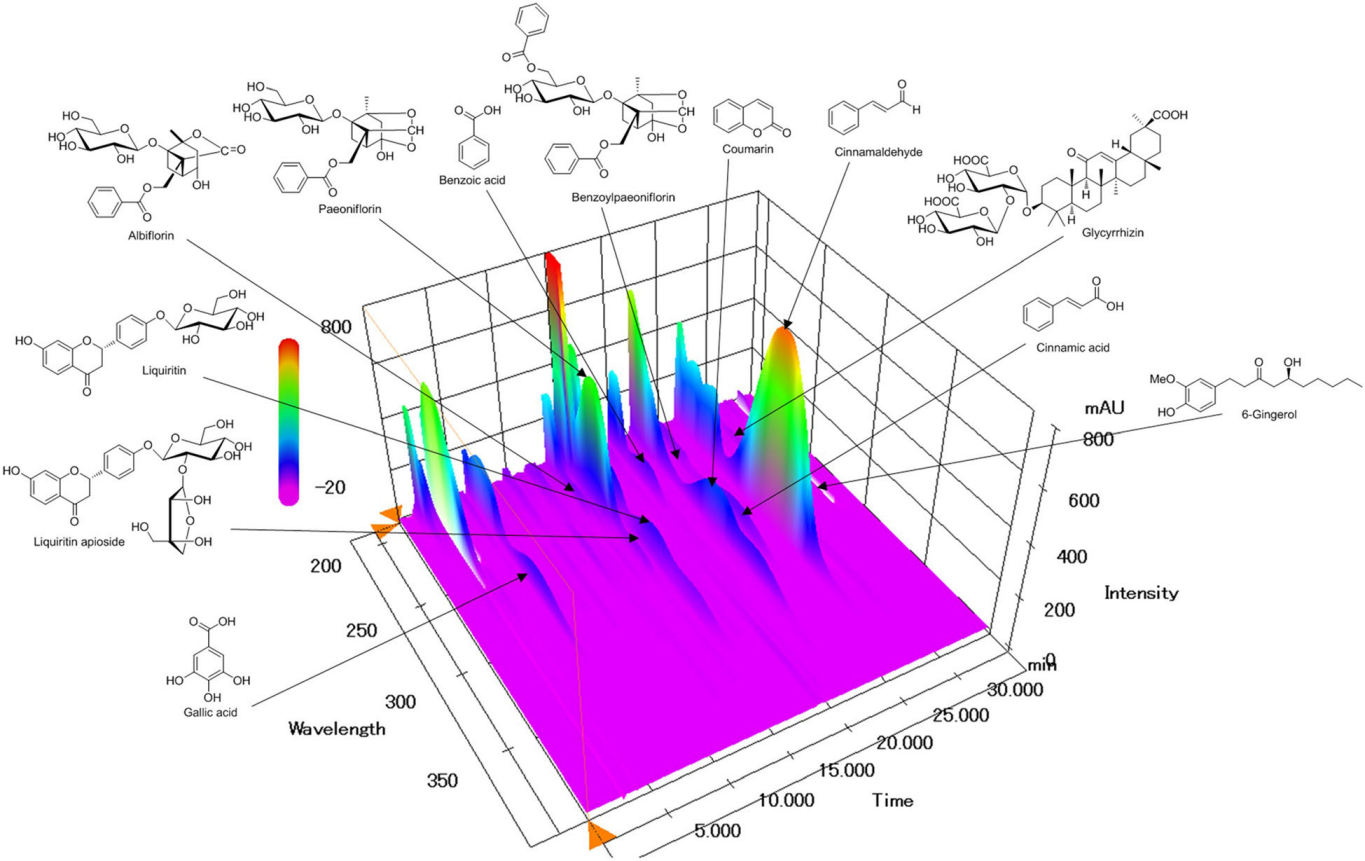
Three-dimensional chromatogram of GJT by HPLC-PDA

All calibration curves were obtained by plotting peak areas versus the concentration of standard solutions in seven different concentration ranges. The calibration curves of the 12 components showed good linearity with correlation coefficients (*r* ^2^) ≥ 0.9997. The limit of detection (LOD) and quantitation (LOQ) were 9.15–258.61 ng/mL and 27.33–783.66 ng/mL, respectively. These results are summarized in Table 2. Using optimized chromatography conditions, three-dimensional chromatograms were obtained using HPLC-PDA detector and 12 compounds were eluted within 35 min (Fig. 6). The amounts of the 12 marker compounds were 0.16–40.29 mg/g and are shown in Table 3.

**Table 2:** Regression data, linear range, correlation coefficient, LOD and LOQ for marker compounds (*n =* 3) of GJT extract

Compound	Linear range (μg/mL)	Regression equation^a^	Correlation coefficient (*r* ^2^)	LOD^b^ (ng/mL)	LOQ^c^ (ng/mL)
Gallic acid	1.56–100.00	*y* = 41971.92× + 834.16	1.0000	45.36	137.44
Albiflorin	1.56–100.00	*y* = 11838.31× + 1993.39	0.9999	197.61	598.81
Paeoniflorin	1.56–100.00	*y* = 14000.91× - 3078.78	1.0000	167.08	506.32
Liquiritin apioside	1.56–100.00	*y* = 16282.26× + 2703.99	0.9999	107.59	326.03
Liquiritin	1.56–100.00	*y* = 18152.25× + 2757.62	0.9999	96.51	292.45
Benzoic acid	1.56–100.00	*y* = 45334.92× + 22784.19	0.9997	51.60	156.37
Coumarin	1.56–100.00	*y* = 49833.92× + 8656.87	0.9999	35.15	106.53
Benzoylpaeoniflorin	0.31–20.00	*y* = 28757.81× + 814.63	0.9999	81.35	246.50
Cinnamic acid	0.31–20.00	*y* = 61499.33× + 3384.72	1.0000	28.49	86.32
Cinnamaldehyde	1.56–100.00	*y* = 131035.51× + 59890.08	0.9997	9.15	27.73
Glycyrrhizin	1.56–100.00	*y* = 8787.34× + 4482.00	1.0000	40.39	122.40
6-Gingerol	0.31–20.00	*y* = 5693.60× + 109.67	1.0000	258.61	783.66

^a^ *y*: peak area (mAU) of compounds; ×: concentration (μg/mL) of compounds

^b^LOD = 3.3 × SD / *S*. (SD is the standard deviation of the blanks and *S* is the slope of the calibration curve)

^c^LOQ = 10 × SD / *S*

**Table 3:** Contents of 12 compounds in the GJT by HPLC (*n =* 3)

Compound	Mean (mg/g)	SD × 10^−1^	RSD (%)	Source^a^
Gallic acid	3.18	0.89	2.79	PR
Albiflorin	2.78	0.42	1.52	PR
Paeoniflorin	40.29	1.17	0.29	PR
Liquiritin apioside	4.94	0.80	1.61	GRR
Liquiritin	6.61	0.26	0.39	GRR
Benzoic acid	2.60	0.30	1.14	PR
Coumarin	5.68	0.11	0.19	CR
Benzoylpaeoniflorin	0.72	0.19	2.58	PR
Cinnamic acid	1.76	0.09	0.54	CR
Cinnamaldehyde	9.32	0.08	0.08	CR
Glycyrrhizin	9.91	0.71	0.71	GRR
6-Gingerol	0.16	0.04	2.41	ZRC

^a^ *PR* Paeoniae Radix, *GRR* Glycyrrhizae Radix et Rhizoma, *CR* Cinnamomi Ramulus, *ZRC* Zingiberis Rhizoma Crudus

## Discussion

Inflammation is part of the abnormal body response caused by physical, biological or chemical stimuli [3]. Nonsteroidal anti-inflammatory drugs (NSAIDs) are the most commonly used for treating inflammatory disorders. NSAIDs target the COX enzyme and include aspirin, ibuprofen, and naproxen. However, long-term use of NSAIDs can trigger severe side effects such as gastric erosion, renal damage, myocardial infarction and asthma exacerbation [19]. Thus, novel therapeutic drugs with higher efficacy and fewer side effects are necessary for the treatment of inflammatory disorders.

In the present study, we investigated whether a traditional herbal formula GJT has anti-inflammatory effects in RAW 264.7 murine macrophages. An inflammatory reaction was induced by LPS treatment according to the previous reports [20, 21]. Consistently, our data revealed that LPS stimulation significantly increased levels of proinflammatory cytokines TNF-α and IL-6 in RAW 264. 7 cells. By contrast, GJT treatment inhibited LPS-induced secretion of TNF-α and IL-6 without cytotoxicity. Anti-inflammatory activity of GJT extract was further confirmed by analyzing the expressions of PGE_2_ and COX-2. Inflammatory stimulators such as LPS can induce COX-2 expression that produces PGE_2_, a key inflammatory mediator [22]. In the present study, GJT extract significantly decreased LPS-stimulated PGE_2_ production and COX-2 mRNA expression in RAW 264.7 cells. HO-1 overexpression inhibits proinflammatory cytokine production and suppresses proinflammatory enzymes [23]. Thus, we investigated whether GJT influence on the expression of HO-1. We found that GJT treatment enhanced protein expression of HO-1 in RAW 264.7 macrophages. However, dose-dependency of HO-1 expression by GJT was consistent with the production of TNF-α, but not IL-6 and PGE_2_. Further experiments will be required to determine the relationship of anti-inflammatory and antioxidant activities of GJP in macrophages.

Molecular regulation of inflammatory responses is closely associated with several signaling pathways such as those of MAPK and NF-κB. Macrophage stimulation with LPS induces phosphorylation of MAPK family proteins ERK1/2, JNK, and p38 MAPK [24]. We observed that LPS stimulation clearly increased levels of phosphorylated ERK1/2, JNK, and p38 MAPK in RAW 264.7 cells. By contrast, GJT extract suppressed phosphorylation of ERK1/2, but not JNK or p38 MAPK, in LPS-stimulated cells, indicating the importance of ERK1/2 to the anti-inflammatory regulation of GJT extract. However, we cannot exclude the possible inhibition of NF-κB activation. Indeed, our immunoblotting and immunofluorescence staining data showed that GJT treatment clearly reduced the nuclear level of NF-κB p65. Overall, GJT potentially controls inflammatory markers by blocking ERK and NF-κB signaling pathways in macrophages.

As mentioned above, GJT consists of 5 different herbal medicines *Cinnamomum cassia*, *Paeonia lactiflora*, *Glycyrrhiza uralensis, Zingiber officinale,* and *Zizyphus jujube* with certain ratio based on ‘Sang han lon’. Of interest, it has been reported anti-inflammatory effects of individual herbs of GJT [25–29]. In addition, the major components of the 5 medicinal herbs are known as follows: coumarins (e.g. coumarin) and phenylpropanoids (e.g. cinnamic acid and cinnamaldehyde) from *C. cassia* [30, 31], monoterpenoids (e.g. albiflorin and paeoniflorin) from *P. lactiflora*, triterpene saponin (e.g. glycyrrhizin) and flavonoids (e.g. liquiritin and liquiritigenin), from *G. uralensis* [32], phenols (e.g. 6-, 8-, and 10-gingerol) from *Z. officinale* [33], and flavonoids (e.g. spinosin and 6ʹʹʹ-feruloylspinosin) from *Z. jujube* [34]. Among those constituents, we analyzed 12 compounds, such as galiic acid, albiflorin, paeoniflorin, liquiritin apioside, liquiritin, benzoic acid, coumarin, cinnamic acid, benzoyl paeoniflorin, cinnamaldehyde, glycyrrhizin, and 6-gingerol using HPLC–PDA. An optimized HPLC–PDA method was applied for quantitative analysis of these 12 compounds in the GJT water extract. Consequently, paeoniflorin (40.29 ± 0.12 mg/g), which is marker compound of *P. lactiflora*, was detected as the most abundant component in GJT extract. Anti-inflammatory activities of several GJT component compounds have been reported, including gallic acid [35], paeoniflorin [36], benzoic acid [37], cinnamaldehyde [38], glycyrrhizin [39], and 6-gingerol [40]. Further studies, including in animal models, will be required to elucidate the precise pharmacological mechanisms of the active compounds from GJT extract and their pharmacokinetics/pharmacodynamics.

## Conclusions

Our results show that an water extract of the traditional herbal formula GJT inhibits LPS-stimulated production of proinflammatory cytokines TNF-α and IL-6, and proinflammatory mediator PGE_2_ in macrophages. Anti-inflammatory activity of GJT is associated with enhancement of HO-1 expression and regulated via targeting ERK and NF-κB pathways. These findings suggest the prophylactic effects if GJT decoction on inflammatory diseases. Further studies will be considered in the near future to determine the effects of GJT for treating inflammatory diseases by the concurrent or post LPS treatment with GJT decoction.
